# Beverage consumption patterns and primary dysmenorrhea among women of reproductive age: a population-based study in Beijing, China

**DOI:** 10.3389/fnut.2026.1797903

**Published:** 2026-05-19

**Authors:** Jian Zhao, Daqian Zhang

**Affiliations:** School of Population Medicine and Public Health, Chinese Academy of Medical Sciences and Peking Union Medical College, Beijing, China

**Keywords:** beverage consumption, caffeine, coffee and tea, epidemiology, menstrual cycle, milk tea, primary dysmenorrhea

## Abstract

**Background:**

Primary dysmenorrhea (PD) is highly prevalent among women of reproductive age and imposes a substantial burden on daily functioning and quality of life. Beverage consumption has been suggested as a potential lifestyle factor related to menstrual pain; however, evidence remains limited and inconsistent. This study aimed to examine the associations between beverages consumption and PD among women of reproductive age.

**Methods:**

This cross-sectional study was conducted as part of the Multilevel Natural Population and Maternal Cohort Study. A total of 1,247 women of reproductive age were recruited in Beijing between September and November 2021. Sociodemographic data and menstrual health status were collected using a structured questionnaire. Consumption of coffee, brewed leaf tea, floral tea, and milk tea, as well as intake patterns during menstrual and non-menstrual periods were assessed using a validated food frequency questionnaire (FFQ). Multinomial logistic regression was performed to examine associations between beverage consumption and PD.

**Results:**

The overall prevalence of PD was 92.9%, with 23.1% of participants reporting moderate-to-severe pain. Associations with dysmenorrhea severity varied by beverage type and level of habitual consumption. Specifically, moderate and high coffee intake were associated with moderate-to-severe dysmenorrhea (*OR* = 2.819, 95% *CI*: 1.419–5.602; *OR* = 3.100, 95% *CI*: 1.398–6.811), whereas high milk tea intake was associated with both mild and moderate-to-severe pain (*OR* = 2.717, 95% *CI*: 1.448–5.101; *OR* = 3.049, 95% *CI*: 1.498–6.207). In contrast, only moderate brewed leaf tea intake, but not higher intake, was associated with moderate-to-severe dysmenorrhea (*OR* = 2.481, 95% *CI*: 1.116–5.519). No significant association was observed for floral tea. Most women reduced intake during menstruation. Compared with unchanged intake, reductions in coffee (*OR* = 2.197, 95% *CI*: 1.280–3.771), or brewed leaf tea (*OR* = 1.943, 95% *CI*: 1.135–3.323), as well as both increased and decreased milk tea intake (*OR* = 3.588, 95% *CI*: 1.559–8.255; *OR* = 2.362, 95% *CI*: 1.375–4.057) were associated with higher pain severity.

**Conclusions:**

Both habitual beverage consumption and menstrual phase-related changes in intake were associated with the severity of PD in this urban Chinese population, suggesting potential links between dietary habits and menstrual health that warrant further investigation.

## 1 Introduction

Primary dysmenorrhea (PD) is defined as cramping abdominal pain during menstruation in the absence of identifiable pelvic organic pathology (1). Epidemiological studies indicate a global prevalence of 45–95%, and in China, the prevalence of PD has been reported to exceed 70%, underscoring its high burden and the need for greater attention (1–4). Common symptoms of PD include lower abdominal pain, back and thigh pain, headaches, and gastrointestinal disturbances, which not only cause marked physical discomfort, but also reduce quality of life, limit daily activities, and lead to absence from school or work (1, 5). PD is primarily attributed to excessive uterine prostaglandin production, increased uterine contractility, systemic inflammatory responses, and central pain sensitization (1). Although these pathophysiological mechanisms are well-described, healthcare seeking behavior and the rate of effective treatment remain low (6, 7).

Accumulating evidence suggests that lifestyle behaviors may play an important role in modulating the risk and severity of PD (8–10). Beverage consumption has emerged as a potential contributor (11). Tea and coffee are among the most consumed beverages worldwide, yet their consumption patterns vary substantially across regions. In China, coffee has increasingly gained popularity in urban areas in recent years, especially among young women (12). Meanwhile, traditional brewed leaf teas such as green and oolong tea remain integral to the dietary culture (13). In addition, milk tea has become a trendy drink among younger people, and floral teas (e.g., rose tea) are often favored by women for their perceived health benefits (14, 15).

At present, studies on the association between beverages and dysmenorrhea are relatively limited and have mostly focused on the association between coffee and PD. Most existing evidence is derived from Western populations, where coffee consumption is relatively common. In contrast, coffee intake is generally lower in China, and population-based evidence from Chinese women remains scarce. Moreover, findings regarding the relationship between coffee intake and dysmenorrhea have been inconsistent. Several studies, mainly conducted among female students, have reported a positive association, with higher or more frequent intake linked to greater symptom reporting (10, 16–19), whereas several studies have found no significant association (20–22). Beyond coffee, evidence concerning other commonly consumed beverages is particularly limited.

In addition, dietary and beverage intake may vary across the menstrual cycle. Previous studies have shown that total energy intake differs by menstrual cycle phase, typically being higher in the luteal than in the follicular phase, with additional with-in-cycle fluctuations (23). Other studies have reported increased food cravings around the onset of menstruation (24, 25). Furthermore, caffeine pharmacokinetics may vary by cycle phase, potentially altering sensitivity to caffeinated beverages (26). Despite these observations, most existing studies have assessed beverage intake at a single time point without distinguishing consumption during menstruation from intake outside the menstrual period, leaving it unclear whether phase-specific intake patterns or changes across the menstrual cycle are associated with dysmenorrhea.

Therefore, this study aimed to examine the associations between beverage intake and PD among women of reproductive age in Beijing, China, with a specific focus on coffee, brewed tea, milk tea, and floral tea. It was hypothesized that phase-specific beverage intake and within-person changes across the menstrual cycle would be associated with dysmenorrhea severity. Beverage consumption during menstruation and outside menstruation was assessed separately, and within-person changes between these two periods were further analyzed in relation to PD severity. The findings may contribute to a better understanding of beverage consumption in relation to menstrual pain and provide evidence relevant to lifestyle-based approaches to menstrual health management among women.

## 2 Methods

### 2.1 Study population

This was a population-based cross-sectional study conducted as part of the Multilevel Natural Population and Maternal Cohort Study, an ongoing population-based cohort that enrolls residents from communities across the Beijing–Tianjin–Hebei region. For the present analysis, we used data from participants recruited in Beijing. Specifically, during the baseline survey conducted between September and November 2021, participants were enrolled through cluster sampling from the physical examination departments of 10 community health service centers located in the six central districts of Beijing. These community health service centers provide routine health examinations to residents within their respective catchment areas, thereby covering a broad segment of the local community population.

The general inclusion criteria for the cohort were: (1) age between 20 and 80 years; (2) residence in the local area for at least 1 year; (3) no major physical disabilities or psychiatric disorders; and (4) not pregnant or postpartum at the time of enrollment. A total of 5,542 participants were initially assessed for eligibility. For the present analysis, we further restricted the sample to women who were premenopausal, yielding a final study population of 1,247 participants. As this study was based on an existing cohort dataset, no *a priori* sample size calculation was performed, and the available sample size was used for all analyses. Participants were recruited during routine examinations at community health service centers. Response rates were not systematically recorded across centers, which precluded formal assessment of non-response bias. The study protocol was reviewed and approved by the Ethics Review Committee of the Institute of Basic Medical Sciences, Chinese Academy of Medical Sciences (Approval No. 067-2021).

### 2.2 Data collection and variables

Data were collected using a structured questionnaire administered during the baseline health examination. Information was obtained on sociodemographic characteristics (age, education level, and monthly personal income), anthropometric measures (weight and height, with body mass index (BMI) calculated as kg/m^2^ and categorized according to Chinese criteria), and reproductive and menstrual characteristics (sexual experience, menstrual cycle regularity, menstrual duration, and menstrual flow).

Beverage consumption, including coffee, brewed leaf tea, floral tea, and milk tea, was assessed using a semi-quantitative food frequency questionnaire (FFQ) adapted from previously validated instruments in Chinese populations (27, 28). Participants reported the frequency of consuming beverages in standard servings (one cup ≈150 ml or one bottled drink ≈300 ml) over the past 6 months. To capture menstrual-related variations, participants were additionally asked to report their beverage intake separately for menstrual and non-menstrual periods. For each participant, changes in intake were determined by comparing consumption during menstrual periods with that during non-menstrual periods, and were categorized as increased, decreased, or no change. For analysis, coffee and tea consumption were further classified into four groups according to the frequency of standard servings consumed: non-consumers (never drink), low intake ( ≤ 3 standard servings per month), moderate intake (1–6 standard servings per week or approximately once per day), and high intake (≥2 standard servings per day).

Dysmenorrhea status and pain severity were assessed based on participants' self-reported experience over the past 6 months. Participants were asked whether they had experienced menstrual pain during this period and, if so, to rate the overall severity of pain according to four predefined categories: no pain; mild pain (pain present but not interfering with daily activities, minimal impact on work, no systemic symptoms, and no need for analgesics); moderate pain (daily activities affected, work performance partially impaired, presence of systemic symptoms, and need for analgesics); and severe pain (marked interference with daily activities and work, prominent systemic symptoms, and poor response to analgesics) (57). For statistical analysis, dysmenorrhea severity was categorized as no pain, mild pain, and moderate-to-severe pain, with the latter combining moderate and severe categories due to limited numbers in the severe group.

### 2.3 Statistical analysis

All analyses were conducted using SPSS version 27.0 (IBM Corp., Armonk, NY, USA). Descriptive statistics were used to summarize participant characteristics and the distribution of dysmenorrhea severity. Chi-square (χ^2^) tests were applied to examine univariate associations between participant characteristics and dysmenorrhea severity. Multinomial logistic regression models were fitted to estimate odds ratios (ORs) and 95% confidence intervals (CIs) for the associations between beverage consumption and dysmenorrhea severity, with “no pain” as the reference category. Multinomial logistic regression models were further applied to explore associations between changes in beverage intake and dysmenorrhea severity. All models were adjusted for potential confounders, including age, education level, monthly personal income, BMI, sexual experience, menstrual cycle regularity, menstrual duration, menstrual flow, and history of gynecological diseases. Multicollinearity was assessed using variance inflation factors (VIF). All VIF values were below 2 except for sexual experience and history of gynecological diseases (*VIF* = 19.04 and 18.69, respectively), indicating substantial collinearity between these two covariates. To evaluate the robustness of the findings, sensitivity analyses were conducted by refitting the models after excluding sexual experience, history of gynecological diseases, or both variables. A two-sided *P* < 0.05 was considered statistically significant.

## 3 Results

### 3.1 Prevalence and severity distribution of dysmenorrhea

Among the 1,247 premenopausal women included in the analysis, 1,159 (92.94%) reported experiencing menstrual pain of any intensity during the past 6 months, corresponding to a 6-month prevalence of dysmenorrhea of 92.94%. Specifically, 88 women (7.06%) reported no pain, 861 (69.05%) reported mild pain, 271 (21.73%) reported moderate pain, and 27 (2.17%) reported severe pain. Given the small number of participants with severe dysmenorrhea, the moderate and severe groups were combined into a single category of moderate-to-severe dysmenorrhea in the subsequent analyses.

### 3.2 Characteristics of the study population and univariate analysis of dysmenorrhea

Table 1 summarizes the sociodemographic, anthropometric, and menstrual characteristics of the 1,247 women of reproductive age and their distribution across different levels of dysmenorrhea severity. Significant differences in dysmenorrhea severity were observed across several participant characteristics.

**Table 1:** Characteristics and univariate analysis of dysmenorrhea among reproductive-age women.

Variables	*N*	%	Dysmenorrhea						χ^2^	*P*
			No pain (*N*, %)		Mild pain (*N*, %)		Moderate-to-severe pain (*N*, %)			
All	1,247		88	7.06	861	69.05	298	23.09		
Age (years)										
< 25	209	16.76	8	9.09	129	14.98	72	24.16	40.066	< 0.001
25-29	256	20.53	9	10.23	173	20.09	74	24.83		
30–34	221	17.72	14	15.91	154	17.89	53	17.79		
35–40	255	20.45	24	27.27	187	21.72	44	14.77		
>40	306	24.54	33	37.50	218	25.32	55	18.46		
Education level										
Junior school or less	50	4.01	9	31.03	31	3.60	10	3.36	14.315	0.026
Senior school	163	13.07	14	48.28	107	12.43	42	14.09		
College or university	916	73.46	3	10.34	635	73.75	219	73.49		
Graduate	118	9.46	3	10.34	88	10.22	27	9.06		
Monthly personal income										
< 5,000	288	23.10	27	30.68	184	21.37	77	25.84	7.565	0.674
5,000–10,000	368	29.51	20	22.73	259	30.08	89	29.87		
10,000–15,000	406	32.56	28	31.82	286	33.22	92	30.87		
>15,000	185	14.84	13	14.77	132	15.33	40	13.42		
BMI										
Marasmus	196	15.72	12	13.64	118	13.70	66	22.15	19.007	0.004
Normal	881	70.65	59	67.05	628	72.94	194	65.10		
Overweight	118	9.46	15	17.05	78	9.06	25	8.39		
Obesity	52	4.17	2	2.27	37	4.30	13	4.36		
Having had a sex life										
Yes	998	80.03	74	84.09	704	81.77	220	73.83	9.709	0.008
No	249	19.97	14	15.91	157	18.23	78	26.17		
Regularity of menstrual cycle										
Regular	952	76.34	79	89.77	670	77.82	203	68.12	20.978	< 0.001
Irregular	295	23.66	9	10.23	191	22.18	95	31.88		
Bleeding duration										
< 3 days	45	3.61	7	7.95	27	3.14	11	3.69	9.331	0.053
3–7 days	1,143	91.66	79	89.77	797	92.57	267	89.60		
>7 days	59	4.73	2	2.27	37	4.30	20	6.71		
Volume of menstrual flow										
Light	174	13.95	10	11.36	120	13.94	44	14.77	16.960	0.002
Normal	866	69.45	67	76.14	616	71.54	183	61.41		
Heavy	207	16.60	11	12.50	125	14.52	71	23.83		
History of gynecological diseases										
Yes	162	12.99	13	14.77	101	11.73	48	16.11	6.814	0.146
No	963	77.23	71	80.68	672	78.05	220	73.83		
Unknown	122	9.78	4	4.55	88	10.25	30	10.07		

Dysmenorrhea severity varied significantly by age group (χ^2^ = 40.066, *P* < 0.001). Younger women were more frequently represented among those reporting moderate-to-severe dysmenorrhea, whereas older age groups accounted for a larger proportion of women reporting no or mild pain. Education level was also significantly associated with dysmenorrhea severity (χ^2^ = 14.315, *P* = 0.026). Women with higher educational attainment constituted a greater proportion of those reporting dysmenorrhea, while women with lower education levels were more commonly represented among those reporting no pain. No significant association was observed between monthly personal income (*P* = 0.674) or history of gynecological diseases (*P* = 0.146) and dysmenorrhea severity.

Dysmenorrhea severity differed significantly across BMI categories (χ^2^ = 19.007, *P* = 0.004). Underweight women accounted for a relatively larger share of moderate-to-severe dysmenorrhea cases compared with their representation in the overall study population, whereas women with normal BMI constituted the majority of mild dysmenorrhea cases. Sexual experience was significantly associated with dysmenorrhea severity (χ^2^ = 9.709, *P* = 0.008). Women with sexual experience comprised a higher proportion of those reporting dysmenorrhea, while women without sexual experience were more frequently represented among those reporting no pain.

Menstrual characteristics were also associated with dysmenorrhea severity. Women with regular menstrual cycles accounted for a greater proportion of those reporting no pain, whereas women with irregular cycles were more frequently represented among those reporting moderate-to-severe dysmenorrhea (χ^2^ = 20.978, *P* < 0.001). No statistically significant differences were observed in dysmenorrhea severity across categories of menstrual duration (*P* = 0.053). Menstrual flow showed a significant association with dysmenorrhea severity (χ^2^ = 16.960, *P* = 0.002). Women reporting heavy menstrual flow constituted a larger proportion of moderate-to-severe dysmenorrhea cases, whereas women with normal menstrual flow were more commonly represented among those reporting no pain or mild dysmenorrhea.

### 3.3 Beverage consumption among women during non-menstrual and menstrual periods and changes between the two phases

**Figure 1:**
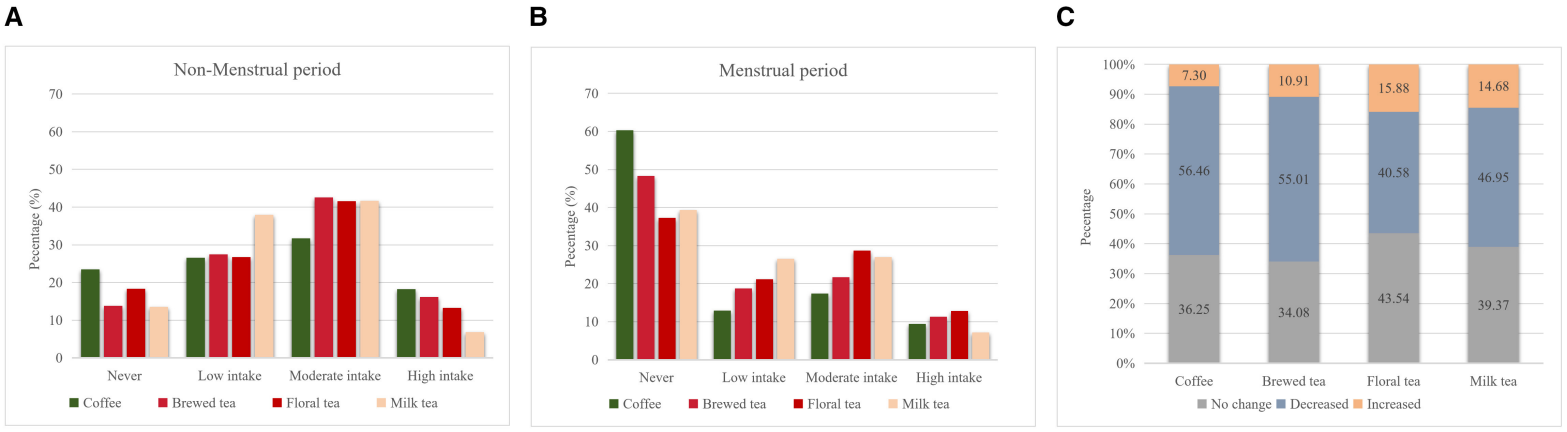
Coffee and tea consumption among women during non-menstrual **(A)** and menstrual periods **(B)** and changes between the two phases **(C)**. Differences in distributions between menstrual and non-menstrual periods were assessed using χ^2^ tests, and all beverages showed significant differences (all *P* < 0.001).

Figure 1 shows the frequency of beverage consumption among reproductive-age women during the non-menstrual (A) and menstrual (B) periods. During the non-menstrual period, coffee was most consumed at a “moderate intake” level (31.76%), followed by “low intake” (26.54%) and “never” consumed (23.50%), with 18.20% reporting high intake. Brewed tea was predominantly consumed at a “moderate intake” level (42.58%), and floral tea and milk tea followed a similar trend, with moderate in-take proportions of 41.54% and 41.70%, respectively.

During the menstrual period, the proportion of women who reported “never” drinking coffee increased markedly to 60.30%, while the proportion reporting high in-take declined to 9.38%. A similar pattern was observed for brewed tea, floral tea, and milk tea, with the proportions of women reporting no consumption increasing to 48.28%, 37.29%, and 39.37%, respectively, accompanied by corresponding decreases in moderate intake. Significant differences in beverage consumption patterns were observed between menstrual and non-menstrual periods for all beverages, including coffee (χ^2^ = 406.3, *P* < 0.001), brewed tea (χ^2^ = 265.1, *P* < 0.001), floral tea (χ^2^ = 113.7, *P* < 0.001), and milk tea (χ^2^ = 163.2, *P* < 0.001).

A within-person comparison among 1,247 women revealed changes in beverage consumption between menstrual and non-menstrual periods (Figure 1). The majority of women decreased their intake during menstruation. Specifically, 56.46% of women reported a decrease in coffee consumption, and 55.01% reduced their intake of brewed tea. Decreases were also reported for floral tea (40.58%) and milk tea (46.95%). In contrast, a smaller proportion of women reported increased consumption during menstruation, including 7.30% for coffee, 10.91% for brewed tea, 15.88% for floral tea, and 14.68% for milk tea. Additionally, a substantial proportion of women reported no change in consumption, with 36.25% maintaining coffee intake, 34.08% maintaining brewed tea intake, 43.54% reporting no change in floral tea consumption, and 39.37% reporting no change in milk tea intake.

### 3.4 Logistic regression analysis of beverage consumption and dysmenorrhea

After adjusting for age, education level, monthly personal income, BMI, sexual experience, menstrual duration, menstrual flow, menstrual cycle regularity, and history of gynecological diseases, multinomial logistic regression analysis revealed significant associations between beverage consumption and the severity of dysmenorrhea (Table 2). For coffee consumption, compared with women who never consumed coffee, moderate intake was significantly associated with higher odds of moderate-to-severe dysmenorrhea (*OR* = 2.819, 95% *CI*: 1.419–5.602, *P* = 0.003). High coffee intake was also significantly associated with moderate-to-severe dysmenorrhea (*OR* = 3.100, 95% *CI*: 1.398–6.811, *P* = 0.005), whereas no significant association was observed with mild dysmenorrhea. For brewed tea, moderate intake was significantly associated with higher odds of moderate-to-severe pain (*OR* = 2.481, 95% *CI*: 1.116–5.519, *P* = 0.026). Low and high intakes were not significantly associated with either mild or moderate-to-severe pain. No significant associations were observed between floral tea consumption and dysmenorrhea severity across all intake levels. For milk tea, high intake was significantly associated with both mild dysmenorrhea (*OR* = 2.717, 95% *CI*: 1.448–5.101, *P* = 0.002) and moderate-to-severe dysmenorrhea (*OR* = 3.049, 95% *CI*: 1.498–6.207, *P* = 0.002). Sensitivity analyses showed that the associations between coffee and milk tea consumption and moderate-to-severe dysmenorrhea remained materially unchanged across models (Supplementary Table S1).

**Table 2:**
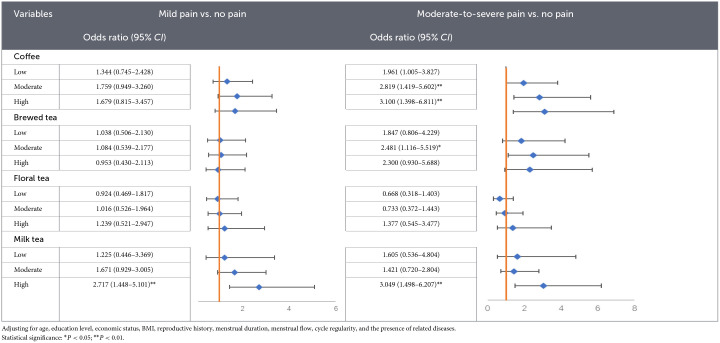
Adaptation to score and Conscientiousness (Medium level included).

### 3.5 Logistic regression analysis of within-person beverage consumption changes and dysmenorrhea

Multinomial logistic regression analyses were conducted to examine associations between within-person changes in beverage consumption between the non-menstrual and menstrual periods and dysmenorrhea severity, adjusting for the same set of potential confounders (Table 3). For coffee, compared with women reporting no change in intake, a decrease in coffee consumption was significantly associated with higher odds of moderate-to-severe dysmenorrhea (*OR* = 2.197, 95% *CI*: 1.280–3.771, *P* = 0.004). No significant association was observed for increased intake. For brewed tea, women who re-ported decreased intake were significantly more likely to report moderate-to-severe dysmenorrhea (*OR* = 1.943, 95% *CI*: 1.135–3.323, *P* = 0.015), whereas no significant association was observed for increased intake. For floral tea, both increased and decreased intake were significantly associated with higher odds of moderate-to-severe dysmenorrhea (*OR* = 2.220, 95% *CI*: 1.024–4.813, *P* = 0.043; *OR* = 1.736, 95% *CI*: 1.010–2.983, *P* = 0.046, respectively). For milk tea, both increased and decreased intake were significantly associated with higher odds of moderate-to-severe dysmenorrhea (*OR* = 3.588, 95% *CI*: 1.559–8.255, *P* = 0.003; *OR* = 2.362, 95% *CI*: 1.375–4.057, *P* = 0.002, respectively). Sensitivity analyses showed that the associations between decreased coffee consumption, decreased brewed tea consumption, and both increased and decreased milk tea consumption and moderate-to-severe dysmenorrhea were materially unchanged across alternative models (Supplementary Table S2).

**Table 3:**
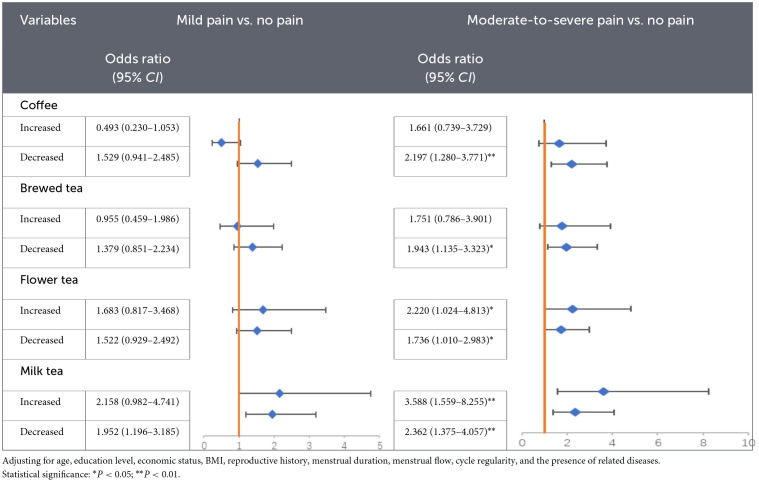
Logistic regression analysis of beverage consumption changes and dysmenorrhea.

## 4 Discussion

### 4.1 High prevalence in this population

The overall prevalence of PD among women of reproductive age in this study was 92.9%, with 23.1% of participants experiencing moderate-to-severe pain. These figures are at the higher end of the prevalence globally reported range (1–3). In Chinese population, a meta-analysis among female college students reported a prevalence of 69.5% (95% *CI*: 59.3–79.6) (4), whereas a hospital-based study in Beijing reported a higher prevalence of 86.4% (29). Compared with these studies, the prevalence of dysmenorrhea observed among women in Beijing in our study was higher. Our observed proportion of moderate-to-severe cases is also broadly comparable to international estimates. A systematic review reported that severe, activity-limiting dysmenorrhea occurs in approximately 2%−29% of women across studies (2). In a population-based sample of 10,070 women, 21.2% reported severe pain in their most recent menstrual cycle (30). The high prevalence and substantial proportion of moderate-to-severe cases in our study may be partly attributed to the higher health awareness and greater willingness to report menstrual pain among urban residents (31, 32). In addition, lifestyle factors commonly observed in urban settings, such as psychosocial stress and irregular sleep, may contribute to the increased risk of dysmenorrhea (33, 34). Moreover, participants were recruited from community health centre during routine examinations, which may over represent individuals with higher health awareness or health concerns, thereby introducing potential selection bias and contributing to the elevated prevalence observed. Together, these findings highlight the significant burden of PD in urban populations and underscore the need for targeted public health interventions and preventive strategies.

### 4.2 Associations between beverage intake and dysmenorrhea severity

The present study found that moderate and high coffee intake were significantly associated with higher odds of moderate-to-severe dysmenorrhea. This finding is consistent with several previous studies that have identified caffeine as a potential risk factor for PD (10, 18, 19, 35). One possible explanation is that caffeine, the main bioactive component of coffee, is a non-selective adenosine receptor antagonist (36). By blocking A1/A2A adenosine receptors, caffeine increases uterine vascular resistance and reduces local blood perfusion (37). This reduction in uterine blood flow may aggravate ischemia-related pain and thereby contribute to more severe dysmenorrhea (56). In addition, previous studies have shown that higher caffeine consumption is associated with alterations in female hormone profiles, including sex hormone-binding globulin (SHBG), estradiol (E2), and total testosterone (TT) (38–40). Given that SHBG, E2, and androgen levels have been directly linked to dysmenorrhea risk and pain se-verity (41–43), these findings suggest that caffeine may influence dysmenorrhea partly through endocrine modulation.

Similarly, high milk tea consumption was also significantly associated with both mild and moderate-to-severe dysmenorrhea. As milk tea typically contains caffeine, it may influence dysmenorrhea through mechanisms similar to those proposed for other caffeine-containing beverages. Moreover, milk tea is commonly a sugar-sweetened beverage and often contains substantial amounts of added sugars and fat. Prior studies have reported that higher intake of sugar-sweetened beverages and diets characterized by frequent consumption of high-fat and sweet foods are associated with increased risk and/or greater severity of primary dysmenorrhea (22, 44). The observed association between high milk tea consumption and more severe menstrual pain may reflect the combined presence of caffeine and added sugars and dairy fat (11).

Interestingly, moderate intake of brewed leaf tea, such as green or oolong tea, was associated with moderate-to-severe dysmenorrhea, whereas high intake was not. This pattern partially aligns with findings from other Chinese cohort studies reporting that tea consumption may have a protective effect against dysmenorrhea (45). Tea leaves are rich in plant-derived phytochemicals such as epigallocatechin gallate (EGCG), a catechin with anti-inflammatory, antioxidant, and smooth muscle relaxing properties, which may help alleviate menstrual pain by modulating prostaglandin metabolism and improving microcirculation (46, 47). At the same time, brewed leaf tea also contains caffeine as its primary methylxanthines, which have stimulatory effects that may contribute to menstrual pain, as discussed in the previous section on caffeine. In our study, only moderate intake, not high intake, was associated with dysmenorrhea severity, suggesting a non-linear pattern. This pattern may reflect a balance between the stimulatory effects of methylxanthines and the potentially protective effects of tea polyphenols. A speculative explanation is that at higher levels of tea consumption, L-theanine, an amino acid unique to tea, may interact with caffeine to attenuate caffeine-induced overstimulation, thereby partially offsetting the stimulatory effects of methylxanthines and modulating pain-related responses (48–50). However, this hypothesis requires further investigation.

Given the limited evidence available from current research, further longitudinal and interventional studies are required to clarify the causal effects of different types of beverages on dysmenorrhea and to elucidate the mechanisms by which specific beverage constituents, such as caffeine, sugars, and tea polyphenols, influence the biological pathways underlying menstrual pain. Such investigations would provide more robust evidence on whether modifying beverage consumption could serve as an effective strategy for the management of menstrual symptoms.

### 4.3 Beverage consumption changes during menstruation and their associations with dysmenorrhea

Marked differences in beverage consumption patterns were observed between menstrual and non-menstrual periods in this study. Overall, most women reported reducing their intake of beverages during menstruation, with the largest decreases observed for coffee and brewed leaf tea. Multivariate logistic regression analyses further showed that, compared with women whose intake remained unchanged, those who reported reduced consumption of coffee or brewed tea were more likely to report moderate-to-severe dysmenorrhea. Given the cross-sectional nature of the data and the assessment of within-person changes, this pattern is consistent with reverse causality (51), whereby women experiencing more severe menstrual pain may consciously limit their intake of caffeine-containing beverages to alleviate symptoms, reflecting behavioral adaptation rather than causal effects of reduced intake (52). In contrast, floral tea and milk tea showed a bidirectional association with dysmenorrhea severity, as both increased and decreased intake were associated with higher odds of moderate-to-severe pain. These findings indicated substantial heterogeneity in behavioral responses during menstruation. These behaviors may be influenced by both physiological experiences and perceptions of beverage-related health effects.

Previous studies have reported that exposure to cold environments or consumption of cold foods and beverages during menstruation may exacerbate menstrual pain or increase dysmenorrhea symptoms (4, 53). Beyond thermal factors, culturally shaped health beliefs may further influence beverage-related behaviors. In the Chinese cultural context, beverages such as coffee, brewed leaf tea, floral tea, and milk tea, are sometimes perceived as “cold” or “stimulating”, regardless of their actual serving temperature (54). As a result, women, particularly those with more severe pain, may reduce intake based on culturally informed expectations of symptom relief (55). At the same time, floral tea and milk tea were also the beverages most increased during menstruation. This apparent contradiction likely reflects divergent interpretations of these drinks. While some women may avoid them due to concerns about irritation or adverse health effects, others may increase consumption because they perceive these beverages as warm, soothing, or emotionally comforting. In the case of milk tea, such perceptions may coexist with limited awareness of its caffeine, added sugar, and dairy fat content, which are components potentially relevant to inflammatory and pain-related pathways. These contrasting behaviors highlight the complexity of self-regulated dietary adjustments during menstruation.

Overall, these findings highlight the complex interplay between cultural norms, individual pain experiences, and self-regulated dietary behaviors. Although such changes represent women's active efforts to manage menstrual discomfort, they also emphasize the need for clearer scientific evidence regarding the physiological effects of different beverages on dysmenorrhea. Future longitudinal and interventional studies are needed to disentangle behavioral adaptation from biological causality, and qualitative or ethnographic research on Chinese menstrual practices would help elucidate the role of cultural factors to support the development of evidence-based dietary guidance for menstrual health management.

### 4.4 Strengths and limitations

This study has several strengths. First, it is among the few population-based studies to simultaneously examine habitual beverage consumption and menstrual cycle-specific intake in relation to dysmenorrhea, providing a more nuanced assessment of beverage-related behaviors. The analysis of within-person changes in consumption between menstrual and non-menstrual periods offers additional insight into behavioral adaptation during menstruation, which has been rarely explored in previous research. Second, the inclusion of multiple beverage types, including coffee, brewed leaf tea, floral tea, and milk tea, allows for a more comprehensive evaluation of commonly consumed drinks within the Chinese dietary context. Third, the adjustment for a wide range of sociodemographic, reproductive, and lifestyle-related confounders enhances the internal validity and robustness of the observed associations.

Several limitations should be considered. First, the cross-sectional design limits the ability to establish temporal or causal relationships, and reverse causality remains a plausible explanation for some associations, particularly those involving changes in beverage intake during menstruation. Second, the restriction of the study sample to urban women in Beijing recruited from community health centers may introduce selection bias and limit external validity, as the findings may not be directly generalizable to populations with different socio-demographic characteristics, healthcare access, or cultural contexts. Third, information on non-respondents was not available, precluding formal assessment of non-response bias. These biases may have contributed to an overestimation of the observed prevalence and potentially influenced the associations; however, their magnitude is difficult to quantify. Fourth, beverage consumption and dysmenorrhea severity were self-reported and assessed at a single time point, which may be subject to recall bias and misclassification. In addition, detailed information on caffeine and sugar content, beverage preparation methods, and portion sizes was not available, and other potential sources of caffeine intake and dietary confounders were not assessed, which may potentially lead to exposure misclassification and residual confounding. Finally, cultural beliefs and perceptions related to beverage consumption during menstruation were not directly measured, although they may have influenced self-regulated dietary behaviors. Future longitudinal analyses within this cohort, together with hybrid studies combining self-report with clinical verification are warranted to clarify the directionality of the associations and reduce misclassification bias.

## 5 Conclusions

In conclusion, this population-based study highlights a high prevalence of primary dysmenorrhea among women of reproductive age in Beijing, underscoring its substantial public health burden in urban settings. The findings suggest that habitual consumption of certain beverages, particularly coffee, brewed leaf tea, and milk tea, is associated with the severity of dysmenorrhea. In addition, beverage consumption patterns were found to vary across the menstrual cycle, and changes in intake between menstrual and non-menstrual periods were also associated with dysmenorrhea severity, reflecting complex interactions between pain experiences and self-regulated dietary behaviors. These results emphasize that both habitual beverage intake and menstrual phase-related behavioral adjustments may be relevant factors in menstrual pain experiences. Although causal inferences cannot be drawn, the study provides novel evidence on beverage-related behaviors in relation to dysmenorrhea within a Chinese cultural context. Future longitudinal and interventional studies are warranted to clarify temporal relationships, elucidate underlying biological mechanisms, and inform evidence-based dietary guidance for menstrual health management.

## Data Availability

The raw data supporting the conclusions of this article will be made available by the authors, without undue reservation.
