# Supplementary material for: Beverage consumption patterns and primary dysmenorrhea among women of reproductive age: a population-based study in Beijing, China
Source: Front Nutr. 2026 May 19;13:1797903. doi: 10.3389/fnut.2026.1797903 (PMC13226207; doi:10.3389/fnut.2026.1797903)
Supplement: Supplementary file 1 [file Table_1.pdf]

Supplementary

Table S1. Sensitivity analyses for associations between beverage consumption and dysmenorrhea severity.

Variables	Main model OR (95% CI)	Model S1[a]	Model S2[b]	Model S3[c]
Coffee (VS Never)				
Moderate	2.819 (1.419–5.602)	2.765 (1.395–5.480)	2.902 (1.462–5.758)	2.701 (1.368–5.332)
High	3.100 (1.398–6.811)	3.045 (1.372–6.754)	3.215 (1.452–7.118)	2.984 (1.341–6.641)
Milk tea (VS Never)				
Moderate	3.049 (1.498–6.207)	2.978 (1.462–6.065)	3.132 (1.537–6.381)	2.915 (1.431–5.936)

a: Model S1: Excluding sexual experience
b: Model S2: Excluding history of gynecological diseases
c: Model S3: Excluding both sexual experience and history of gynecological diseases

Table S2. Sensitivity analyses for associations between changes in beverage consumption during menstruation and moderate-to-severe dysmenorrhea.

Variables	Main model OR (95% CI)	Model S1[a]	Model S2[b]	Model S3[c]
Coffee (VS No change)				
Decreased	2.197 (1.280–3.771)	2.143 (1.251–3.672)	2.231 (1.299–3.830)	2.105 (1.228–3.609)
Brewed tea (VS No change)				
Decreased	1.943 (1.135–3.323)	1.901 (1.110–3.255)	1.978 (1.154–3.390)	1.864 (1.089–3.192)
Milk tea (VS No change)				
Increased	3.588 (1.559–8.255)	3.472 (1.510–7.984)	3.641 (1.582–8.381)	3.401 (1.478–7.828)
Decreased	2.362 (1.375–4.057)	2.294 (1.334–3.946)	2.417 (1.406–4.156)	2.251 (1.308–3.873)

a: Model S1: Excluding sexual experience

b: Model S2: Excluding history of gynecological diseases

c: Model S3: Excluding both sexual experience and history of gynecological diseases
